# Congenital Metabolic Bone Disorders as a Cause of Bone Fragility

**DOI:** 10.3390/ijms221910281

**Published:** 2021-09-24

**Authors:** Francesca Marini, Francesca Giusti, Teresa Iantomasi, Maria Luisa Brandi

**Affiliations:** 1 Department of Experimental and Clinical Biomedical Sciences, University of Florence, 50139 Florence, Italy; francesca.marini@unifi.it (F.M.); francesca.giusti@unifi.it (F.G.); teresa.iantomasi@unifi.it (T.I.); 2 F.I.R.M.O. Fondazione Italiana per la Ricerca sulle Malattie dell’Osso, Italian Foundation for the Research on Bone Diseases, 50141 Florence, Italy

**Keywords:** congenital metabolic bone disorders, skeletal development, bone turnover, mineral metabolism, bone mineralization, bone fragility, pathological fractures

## Abstract

Bone fragility is a pathological condition caused by altered homeostasis of the mineralized bone mass with deterioration of the microarchitecture of the bone tissue, which results in a reduction of bone strength and an increased risk of fracture, even in the absence of high-impact trauma. The most common cause of bone fragility is primary osteoporosis in the elderly. However, bone fragility can manifest at any age, within the context of a wide spectrum of congenital rare bone metabolic diseases in which the inherited genetic defect alters correct bone modeling and remodeling at different points and aspects of bone synthesis and/or bone resorption, leading to defective bone tissue highly prone to long bone bowing, stress fractures and pseudofractures, and/or fragility fractures. To date, over 100 different Mendelian-inherited metabolic bone disorders have been identified and included in the OMIM database, associated with germinal heterozygote, compound heterozygote, or homozygote mutations, affecting over 80 different genes involved in the regulation of bone and mineral metabolism. This manuscript reviews clinical bone phenotypes, and the associated bone fragility in rare congenital metabolic bone disorders, following a disease taxonomic classification based on deranged bone metabolic activity.

## 1. Introduction

Bone is a mineralized connective tissue (hard tissue), which exerts important biological functions, such as locomotion, support, and protection of soft tissues and organs, as well as being the storage of calcium and phosphate [1].

Despite its inert appearance, bone is a highly active tissue, continuously undergoing a remodeling process, by which the old tissue is replaced by new bone, granting the skeleton the ability to adapt to mechanical use, correct calcium and phosphate homeostasis, and to heal fractures. The correct equilibrium between bone resorption and new bone formation is necessary for skeletal health. An imbalance between these two phases results in bone fragility, a pathological condition in which the correct bone microarchitecture is altered, the strength of bone tissue is reduced, and the skeleton is prone to deformities and fractures, even in the presence of low-impact traumas or with no trauma [2].

Skeletal development and life-long bone turnover are two finely and complexly regulated processes, in which numerous local and systemic factors participate (chemokines, cytokines, hormones, intracellular signals, and biomechanical stimulation) [3,4]. A variety of genes and epigenetic factors concur for the correct modeling and remodeling of the skeleton. As a consequence, a defect of expression and/or activity in one of these key factors can alter normal bone turnover and be responsible for bone fragility.

At the cellular level, bone fragility can be caused by excessive osteoclast-driven bone resorption that is not balanced by a corresponding amount of bone formation, which leads to bone mass loss and “porous bone” (osteoporosis), or by disfunctions specifically affecting the correct mineralization process of the extracellular matrix leading to “soft bone” (a pathological condition named osteomalacia in adults and rickets in children), or by an excessive bone mass (osteopetrosis) being the outcome of an enhanced osteoblast-driven mineralized bone deposition or a reduced resorption activity by the osteoclasts. Despite their different molecular causes and histological manifestations, these bone pathological conditions confer an elevated rate of deformities to the tissue and notably increase the risk of fragility fractures. In osteoporosis and osteomalacia, bone fragility is caused by quantitatively low bone mass or by poorly mineralized bone, respectively. Conversely, in osteopetrosis, bone fragility can be caused by excessive bone formation and mineralization density, which, rather than conferring additional strength, lead to a lack of normal tissue turnover and bone repair, with consequent structural brittleness, predisposing the bone to fracture [5].

Clinically, the most common cause of bone fragility is idiopathic osteoporosis of the elderly. Aging is the main cause of progressive bone mass reduction, acting in synergy with pre-existent endogenous (genetic and epigenetic signatures) and exogenous (lifestyle and diet) risk factors. Osteoporosis is defined, according to the World Health Organization criteria, as a bone mineral density (BMD) value that is more than 2.5 standard deviations below that of the mean level for a young adult reference population [6]. Fragility fractures, occurring prevalently at wrists, vertebrae, and proximal femur, but also at ribs and humerus, represent the clinical endpoint of this pathological condition. Osteoporosis can also manifest as a secondary consequence to a varied spectrum of diseases, affecting organs other than the skeleton, which alter mineral metabolism, and indirectly, correct bone homeostasis [7].

In addition, bone fragility can manifest at any age, as a consequence of a wide spectrum of rare congenital metabolic bone disorders, in which the inherited genetic defect compromises the correct bone tissue modeling and remodeling, causing bone deformities and fragility fractures.

## 2. Bone Fragility in Rare Congenital Metabolic Bone Disorders

The most recent taxonomic classification of human rare congenital skeletal metabolic diseases, prepared by the Skeletal Rare Diseases Working Group of the International Osteoporosis Foundation, and based on the genetic defect and the deranged bone metabolic activity causing the disease, reported a total of 116 Mendelian-inherited clinical phenotypes, and 86 mutated causative genes, involved in the regulation of bone and mineral metabolism homeostasis [8]. According to this taxonomy, congenital metabolic bone diseases can be divided into four major groups, based on their primary pathogenic molecular mechanisms: (1) disorders due to altered activity of bone cells (osteoclasts, osteoblasts, or osteocytes); (2) disorders due to altered bone extracellular matrix proteins; (3) disorders due to altered bone microenvironmental regulators; and (4) disorders due to altered activity of calciotropic and phosphotropic hormones/regulators.

Inheritance is variable among diseases; it can be autosomal dominant, autosomal recessive, or in rare cases, follows X-linked modes. Mutations are usually inherited from one or both parents; however, more rarely, they may occur de novo at the embryo level [9]. They can be inactivating mutations, leading to a loss-of-function of the encoded protein, or activating mutations, resulting in a gain-of-function of the encoded protein.

### 2.1. Bone Fragility in Bone Disorders Due to Altered Activity of Bone Cells

Bone turnover is a multiphase process that, to develop correctly, requires the coordinated actions of bone cells (osteoblasts, osteoclasts, osteocytes, and bone lining cells). Osteoblasts are the active bone-forming cells that differentiate, under the induction of specific systemic and local signals, from the mesenchymal stem cells of the bone marrow. They are responsible for the secretion of bone extracellular matrix proteins and the promotion of matrix mineralization during the bone structuring and restructuring processes [10]. Osteoclasts are the sole bone-resorbing cells, designed to remove old bone tissue in order to initiate normal bone remodeling and to reabsorb dead bone ends at the fracture site during bone healing. They are multinucleate cells deriving from circulating precursors of the monocyte/macrophage lineage upon stimulation of two essential factors: the monocyte/macrophage colony-stimulating factor (M-CSF) and the receptor activation of NF-κB ligand (RANKL) [11]. Osteocytes, the most abundant bone cell type, are mature osteoblasts embedded within calcified bone matrix, which act as mechano-sensors and orchestrators of the bone remodeling process [12]. The function of bone lining cells is not clear, but they seem to play a key role in coupling bone resorption to bone formation [13]. Bone remodeling consists of three sequential phases: (1) an osteoclast-driven initiation of bone resorption, (2) a transition period from resorption to new bone formation, and (3) an osteoblast-driven new bone formation [14].

Alterations in number, differentiation, and/or activity of bone cells are causes of abnormal bone tissue homeostasis. Disorders caused by genetic defects altering the correct functions of bone-forming and bone-reabsorbing cells consist of numerous different rare phenotypes (Table 1), which can be further divided into four subgroups: (1) diseases characterized by low bone resorption (Table 1, Subgroup 1a), (2) diseases characterized by high bone resorption (Table 1, Subgroup 1b), (3) diseases characterized by low bone formation (Table 1, Subgroup 1c), and (4) diseases caused by high bone formation (Table 1, Subgroup 1d).

Diseases characterized by low bone resorption are caused by a reduced osteoclast number and/or a decreased osteoclast function, due to germinal mutations in genes regulating either osteoclast differentiation (*TNFRSF11A*, *TNFSF11*) or osteoclast activity (*CA2*, *CLCN7*, and *CTSK*) [8]. This subgroup includes various phenotypes that, despite their different causative gene defects, share common skeletal characteristics, such as a generalized high bone mass, an increased bone density, and hardening of bone tissue, consisting of thickening of trabecular bone (osteosclerosis) and widening of cortical bone (hyperostosis), which can manifest as solitary sclerotic bone lesions or as diffuse bony sclerosis. As a consequence, these diseases show a high fragility fracture rate, prevalently manifesting in the severe recessive forms.

Conversely, diseases characterized by high bone resorption are caused by a pathologically enhanced osteoclast function. The increased resorptive activity of osteoclasts, not balanced by sufficient formation of new bone tissue, leads to osteoporosis and osteolytic lesions, skeletal deformities and functional impairment, bowed long bones, and a high tendency of pathological fractures [8].

The cause of diseases characterized by high bone mass formation is enhanced activity of osteoblasts resulting in increased mineralized bone mass deposition and increased bone density. This class of diseases includes various clinical phenotypes, mainly caused by mutations in genes regulating osteoblast differentiation from their mesenchymal precursors (*RUNX2*, *LRP5*, *AMER1*, and *LEMD3*) or modulating the activity of mature osteoblasts (*SOST*). Some diseases of this subgroup manifest skeletal overgrowth and deformities, and disease-specific localized bone defects, and, in rare cases, ectopic exostosis. Fragility fractures are rarely reported [8].

Diseases characterized by low bone formation include clinical phenotypes, caused by genetic defects responsible for reduced function of osteoblasts (inactivating mutations in genes necessary for the correct osteoblast differentiation, such as *LRP5*, *RUNX2*, *SP7*, *NOTCH2*, and genes regulating the osteoblast-driven mineralization, such as *IFITM5* and *PLS3*). This subgroup also includes five clinical phenotypes of *Osteogenesis imperfecta* (types V, VI, XII, XV, and XX), not molecularly affecting the structure of collagen type 1 directly, but showing a defective osteoblast activity and/or bone matrix mineralization, resulting in short stature, hypomineralized skeleton, bone deformities, and pathological fractures.

In 1999, Dinolus et al. [21] described a unique inherited bone condition in a three-generation family, presenting expansile bone striatal bilateral lesions of the distal radius and ulna, cortical thickening of the proximal long bones, metaphyseal cupping of the metacarpals and phalanges, and pathologic fractures. The clinical phenotype is currently reported in the OMIM database as “expansile bone lesions” (MIM number 603439), but the genetic cause is still unknown. Cortical thickness, shown in the affected members, and bone phenotype partially overlapping with familial expansile osteolysis suggest that this disease may be caused by an altered activity of bone cells. Inheritance appears to be autosomal dominant.

### 2.2. Bone Fragility in Bone Disorders Due to Altered Extracellular Matrix Proteins

Bone extracellular matrix is composed of inorganic elements (minerals and water) and an organic component (collagen, non-collagenous proteins, and lipids). The correct composition of the matrix is fundamental for the microarchitecture of bone tissue, bone strength and function, and concurs with the regulation of proper matrix mineralization. Collagen type 1 is the most abundant protein (over 90% of the organic matrix) of bone extracellular matrix, and one of the major constituents implicated in its correct mineralization [22]. Therefore, disruption of the correct quantitative and qualitative collagen synthesis and assembly is responsible not only for altered composition of the organic component of bone matrix, but also for defective mineralization, both leading to bone fragility.

Collagen type 1 consists of three post-translationally modified chains, which form a triple helical fibril of two identical α1 chains, and one, structurally similar but genetically different, α2 chain, encoded by the *COL1A1* and *COL1A2* genes, respectively. About 85–90% of patients with inherited diseases caused by alteration of collagen type 1 quantity or structure have an inactivating mutation in one of these two genes [23].

Currently, all the known inherited diseases of the bone matrix affect collagen type 1. These can be divided into the following subgroups: (1) disease caused by genetic defects affecting the collagen type 1 synthesis and structure (Table 2, Subgroup 2a), (2) disease caused by gene mutations altering the post-translational collagen modification (Table 2, Subgroup 2b), and (3) diseases caused by gene mutations involved in the processing and crosslink of collagen (Table 2, Subgroup 2c). All together, these diseases include 16 genetically heterogeneous clinical forms of *Osteogenesis imperfecta*, Bruck syndromes type 1 and type 2 (caused by loss-of-function mutations in two genes encoding proteins involved in the regulation of folding and crosslinking of procollagen type 1), and two *Osteogenesis imperfecta*-like syndromes (Cole-Carpenter syndromes type 1 and type 2) [8]. Despite these clinical forms distinguished by their clinical severity, bone characteristic features commonly overlap. People with these conditions have fragile bones, prone to deformities, that fracture easily, often from a mild trauma or with no apparent cause. Additional pathognomonic bone features may include short stature, curvature of the spine (scoliosis), joint deformities (contractures), and dentinogenesis imperfecta. The severe forms show marked growth deficiency and multiple fractures that may occur even before birth. Conversely, patients with milder forms are usually of normal or near normal height, and show only a few fractures during their lifetime, manifesting prevalently during childhood and adolescence as the result of minor trauma.

### 2.3. Bone Fragility in Bone Disorders Due to Altered Bone Microenvironmental Regulators

The regulation of bone remodeling is both systemic and local. Local regulation of bone homeostasis includes cytokines and growth factors that modulate bone cell functions, or enzymes involved in the control of bone and mineral metabolism, such as alkaline phosphatase (ALP).

According to the genetic defects affecting the bone microenvironmental regulators, these disorders can primarily be divided into the following subgroups: (1) diseases due to altered ALP activity (Table 3, Subgroup 3a), and (2) diseases due to alterations in bone-regulating cytokines and growth factors [8]. The latter can be further divided into: (1) diseases due to alterations of the RANK/RANKL/OPG system (Table 3, Subgroup 3b), (2) diseases due to alterations of the glycosylphosphatidylinositol (GPI) biosynthesis pathway (Table 3, Subgroup 3c), (3) diseases due to alterations of LRP5-Wnt signaling (Table 3, Subgroup 3d), and (4) diseases due to alteration of the bone morphogenetic protein receptor (BMPR) (Table 3, Subgroup 3e).

ALPs are membrane-bound enzymes that hydrolyze monophosphate esters in the presence of an alkaline microenvironment (pH 8–10), releasing inorganic phosphate molecules, necessary for the formation of hydroxyapatite crystals and bone matrix mineralization, and, at the same time, hydrolyzing the inorganic pyrophosphate, one of the main biological inhibitors of bone mineralization [31]. There are four different ALP enzymes in humans, encoded by four different genes: tissue-nonspecific ALP (TNSALP), intestinal, placenta, and germ cell specific isoforms [32]. TNSALP, encoded by the *ALPL* gene, is prevalently expressed in liver, bone, and kidneys, and it accounts for approximately 95% of total serum ALP activity. Disorders of ALP activity are caused by a reduced/absent ALP function (hypophosphatasia, HPP), due to inactivating mutations of the *ALPL* gene, and are characterized by hypomineralization of hard tissues. HPP includes six different clinical forms, i.e., perinatal lethal, prenatal benign, infantile, childhood, adult, and odonto-HPP, following a classification prevalently based on the age of diagnosis and associated with a progressively decreasing degree of severity, ranging from a perinatal lethal form, with absolutely no skeletal mineralization and severe bone deformities, multiple pathological fractures and craniosynostosis, to mild forms, with late adult onset, in which bone fragility manifests principally as early-onset nontraumatic fractures, a delay in fracture healing, recurrent and/or slow-to-heal metatarsal or tibial stress fractures, and unilateral or bilateral subtrochanteric or diaphyseal femoral pseudofractures (atypical femur fractures, AFFs).

RANKL, expressed on the membrane of osteoblast-lineage cells, is the master inductor of differentiation of mature osteoclasts from their hematopoietic precursors [33] through its direct bond with the RANK receptor expressed, in response to M-CSF, on the surface of osteoclast precursor cells. RANKL-RANK signaling is negatively regulated by osteoprotegerin (OPG), which is a soluble decoy receptor for RANKL that prevents the RANKL binding to RANK and inhibits osteoclastogenesis [34]. The five diseases caused by gene mutations altering the RANK/RANKL/OPG system (Table 3, Subgroup 3b) are included in the diseases caused by an altered activity of osteoclasts, two diseases are caused by reduced osteoclast function (Table 1, Subgroup 1a) and three diseases are caused by increased osteoclast function (Table 1, Subgroup 1b).

GPI is a cell surface glycolipid that anchors over 150 proteins (enzymes, receptors, and adhesion molecules) to the cell membrane, concurring to signal transduction [35]. Twenty-two phosphatidyl inositol glycan (PIG) genes are involved in the synthesis of GPI within the endoplasmic reticulum, and four post-GPI attachment to protein (PGAP) genes modify GPI in the endoplasmic reticulum and Golgi [36]. Germinal biallelic mutations in some of these genes have been associated with congenital GPI deficiencies and with a class of diseases known as hyperphosphatasia with mental retardation syndrome (HPMRS), characterized by cognitive delay, intellectual disability, epilepsy, and markedly elevated serum activity of total ALP, leading to typical skeletal abnormalities.

The low-density lipoprotein-related receptor 5 (LRP5) participates in the stabilization and activation of β-catenin, positively regulating the Wnt signaling, which regulates nearly all aspects of osteoblast function, from initial osteogenic lineage commitment to the control of osteoblast differentiation [37]. Genetic alterations of LRP5-Wnt signaling disrupt the correct osteoblastogenesis; inactivating mutations lead to diseases characterized by low bone mass, while activating mutations cause diseases with high bone mass. The five diseases caused by gene mutations altering the LRP5-Wnt signaling (Table 3, Subgroup 3d) are also included in diseases caused by an altered activity of osteoblasts, three diseases are caused by reduced osteoblast function (Table 1, Subgroup 1c) and two diseases are caused by increased osteoblast function (Table 1, Subgroup 1d).

Bone morphogenetic proteins (BMPs) are multifunctional growth factors that play an important role in postnatal bone formation, acting via their bond to serine/threonine kinase transmembrane receptors [38]. To date, only one clinical phenotype has been associated with a heterozygote germinal activating mutation in the Activin A receptor type 1 (*ACVR1*) gene, a component of the BMP receptor (BMPR), which is responsible for fibrodysplasia ossificans progressiva (FOP), an extremely severe and incurable, spontaneously arisen, progressive heterotopic ossification of soft tissues, mainly muscles and tendons.

### 2.4. Bone Fragility in Bone Disorders Due to Altered Activity of Calciotropic and Phosphotropic Hormones/Regulators

Calcium ion and phosphate are the two components of hydroxyapatite crystals of bone mineralized matrix. The appropriate regulation of calcium ion and phosphate homeostasis and their correct availability are fundamental aspects for the mineralization process to properly take place. Calciotropic and phosphotropic hormones are the endocrine effectors regulating the systemic homeostasis of calcium and phosphate, respectively. Calciotropic hormones include the parathyroid hormone (PTH) and the active form of vitamin D (1,25-dihydroxyvitamin D), while the only phosphotropic hormone is the fibroblast growth factor 23 (FGF23).

Diseases affecting the correct regulation of calcium and/or phosphate homeostasis, and, subsequently, bone mineralization, can be classified into: (1) disorders due to an excess or a deficiency of PTH secretion by the parathyroid glands (named hyperparathyroidism and hypoparathyroidism, respectively); (2) disorders caused by abnormal PTH receptor signaling (pseudohypoparathyroidism); (3) disorders due to altered vitamin D metabolism and activity (Table 4); and (4) congenital disorders of the phosphate homeostasis (Table 5).

Inherited forms of primary hyperparathyroidism, due to hyperfunction, hyperplasia, adenoma, or, in extremely rare cases, carcinoma of parathyroid gland(s), which cause an excessive secretion of PTH and persistent hypercalcemia, can occur as isolated diseases (familial isolated primary hyperparathyroidism, familial hypocalciuric hypercalcemia disorders, and neonatal severe hyperparathyroidism), or in the context of congenital endocrine syndromes (multiple endocrine neoplasia syndromes type 1, 2a, and 4, and hyperparathyroidism jaw-tumor syndrome). Persistently elevated PTH induces constant bone resorption, leading to early-onset osteopenia/osteoporosis, both at trabecular and cortical bones, with respect to the reference population of the same age and sex, conferring an increased risk of osteoporotic fragility fractures.

Conversely, congenital forms of primary hypoparathyroidism are a varied group of genetically distinct endocrine disorders, caused by reduced function of the parathyroid glands, characterized by low levels of PTH and hypocalcemia, leading to specific bone signs, such as an increase in trabecular bone volume and cortical bone thickness [39]. Although people with hypoparathyroidism experience an increase in bone mass and are expected to have a reduced rate of fractures, a study by Chawla et al. showed a greater prevalence of vertebral fractures in patients with hypoparathyroidism, especially in postmenopausal women [40]. In addition, a recent study by Starr et al. [41] showed, via a micro-indentation analysis, that individuals with hypoparathyroidism had significantly lower scores of bone material strength index than control subjects, concluding that, despite a “thicker” bone, bone matrix properties are abnormal in hypoparathyroidism, being suggestive of a reduced bone turnover and an increased risk of fractures.

Diseases caused by a deregulated function of PTH develop, in the presence of a normal activity of parathyroids and a correct regulation of PTH synthesis and secretion, as a consequence of genetic defects affecting PTH receptor signaling. They comprise extremely rare forms of congenital pseudohypoparathyroidism caused by tissue resistance to PTH, collectively named “inactivating PTH/PTHrP signaling disorder” (iPPSD), all of which manifest skeletal alterations, ranging from hyperostosis, osteosclerosis, osteodystrophy, and heterotopic ossifications of soft tissues [42].

Genetic disorders that alter correct vitamin D metabolism and function cause defects of growth plates and generally reduced bone mineralization, leading to rickets in children and osteomalacia in adults, both associated with long bone deformities and an increased rate of fragility fractures (Table 4).

Pathological uncontrolled deficiency or excess of serum phosphate concentration are responsible for severe pathologies, secondarily affecting skeleton mineralization. Diseases of the phosphate homeostasis include hypophosphatemic disorders (Table 5, Subgroup 5a) and hyperphosphatemic disorders (Table 5, Subgroup 5b), and are caused by mutations in genes encoding a class of proteins, named phosphatonins, which are responsible for the regulation of phosphate homeostasis [43], such as the FGF23 hormone, the regulators of active FGF23 levels (*GALNT3* and *PHEX*) and activity (*KLOTHO*), the FGF23 receptor (*FGFR1*), and kidney sodium/phosphate cotransporters (NPTIIa and NPTIIc). Hypophosphatemic disorders are characterized by low serum levels of phosphate, hyperphosphaturia (excessive urinary excretion of phosphate), and high serum levels of bone ALP, causing poor bone mineralization (rickets/osteomalacia). Total calcium and calcium ion are normal. Conversely, hyperphosphatemic disorders present high serum levels of phosphate, hypophosphaturia, and elevated serum levels of active vitamin D, causing altered skeletal mineralization and low/normal bone mass, as well as ectopic calcification of soft tissues.

## 3. Conclusions

Although osteoporosis represents the most common cause of pathological fractures, bone fragility is also a common hallmark of a large spectrum of rare congenital metabolic bone disorders, caused by germinal mutations in genes involved in various aspects of regulation of cellular and molecular homeostasis of bone tissue.

To date, over 100 different congenital metabolic bone disorders involving abnormalities of cartilage and bone have been reported, with skeletal phenotypes often overlapping among these rare conditions. As a consequence, a differential diagnosis may require a thorough medical evaluation, including personal and family medical histories, anthropometric evaluation, radiological imaging, biochemical measurements, and genetic counseling, carried out by specialists with specific expertise. The identification of the precise causative genetic variant is of key importance for the diagnosis and clinical management of the patient, since knowing the deregulated pathway(s) responsible for disease development may help personalize clinical care, to choose a specific medical treatment, if available, and to determine the eligibility of the patient to participate in clinical trials underway for novel target therapies.

Multigenic panel testing using next-generation sequencing technique, which allows the simultaneous screening of genes responsible for congenital metabolic bone disorders, including the high-resolution analysis of copy number variants, can provide rapid and comprehensive diagnostic and therapeutic benefits to clinicians and patients, and therefore should become part of the medical work-up for patients.

## Figures and Tables

**Table 1:** Congenital metabolic bone disorders due to altered activity of bone cells.

Disease Name	OMIM Phenotype Number	Causative Gene (Type of Mutations)	Inheritance	Gene Role	Reported Skeletal Phenotypes
**1a. Diseases Characterized by Low Bone Resorption (Reduced Osteoclast Number and/or Function)**					
Osteopetrosis autosomal dominant type 2 (OPTA2)	#166600	*CLCN7* (heterozygote, loss-of-function)	AD	This gene encodes the chloride channel 7 (CLCN7), important for the acidification of the osteoclast resorption lacuna.	Segmentary osteosclerosis, predominantly at the vertebral endplates, iliac wings, and skull base; osteomyelitis, especially of the mandible; osteoarthritis of the hip; and nontraumatic fractures, particularly of the long bones
Osteopetrosis autosomal recessive type 1 (OPTB1)	#259700	*TCIRG1* (homozygote or compound heterozygote, loss-of-function)	AR	This gene encodes a subunit of the vacuolar H(+)-ATPase, regulating the intracellular pH of osteoclasts.	Macrocephaly and frontal bossing, manifesting during the first few months of life; defective remodeling of skull bones, leading to blindness, deafness, and facial palsy, and to choanal stenosis with concomitant feeding difficulties and respiratory problems; abnormal expansion of cortical and trabecular bone that reduces medullary space and leads to bone marrow insufficiency
Osteopetrosis autosomal recessive type 2 (OPTB2)	#259710	*TNFSF11* (homozygote, loss-of-function)	AR	This gene encodes RANKL, the ligand of RANK, which induces osteoclastogenesis.	Mandibular osteomyelitis and prognathism; dental anomalies, metaphyseal modeling defects; bone deformities; poor bone remodeling; tendency to fracture
Osteopetrosis autosomal recessive type 3 (OPTB3)	#259730	*CA2* (homozygote or compound heterozygote, loss-of-function)	AR	This gene encodes the carbonic anhydrase II, an enzyme that is expressed at high levels in osteoclasts during bone resorption.	Short stature; dental malocclusion; early fractures
Osteopetrosis autosomal recessive type 4 (OPTB4)	#611490	*CLCN7* (homozygote or compound heterozygote, loss-of-function)	AR	This gene encodes the chloride channel 7 (CLCN7), important for the acidification of the osteoclast resorption lacuna.	Loss of trabecular structure; poor/no definition between cortical and medullary bone; oblique fractures
Osteopetrosis autosomal recessive type 5 (OPTB5)	#259720	*OSTM1* (homozygote, loss-of-function)	AR	This gene encodes a transmembrane protein required for osteoblast maturation and function. OSTM1 protein colocalizes with CLCN7 in the ruffled border of bone-resorbing osteoclasts, maybe concurring to the acidification of the resorption lacuna.	Loss of trabecular structure; poor/no definition between cortical and medullary bone; generalized osteosclerosis; densely sclerotic fragile bones prone to fractures; progressive obliteration of the marrow spaces leading to bone marrow insufficiency or failure; fracture may manifest in utero
Osteopetrosis autosomal recessive type 6 (OPTB6)	#611497	*PLEKHM1* (homozygote, loss-of-function)	AR	This gene encodes a large, multi-modular, adapter protein, which is implicated in osteoclast vesicle trafficking and bone resorption.	Cortical sclerosis of the pelvis bone; deformity of long bones; chondrolysis of the hip; pain at walking; band-like sclerosis of vertebras, and at the metadiaphyses of the distal femora, tibiae, and fibulae, and proximal fibulae and tibiae
Osteopetrosis autosomal recessive type 7 (OPTB7)	#612301	*TNFRSF11A* (homozygote or compound heterozygote, loss-of-function)	AR	This gene encodes the RANK receptor, expressed on the membrane of osteoclast precursors and one of the main positive regulators of osteoclastogenesis.	Severe osteoclast-poor osteopetrosis; extensive trabecular structures, with retention of large areas of cartilage
Osteopetrosis autosomal recessive type 8 (OPTB8)	#615085	*SNX10* (homozygote, loss-of-function)	AR	This gene encodes the sorting nexin 10 protein which is involved in intracellular vesicular trafficking, essential for osteoclast resorption activity.	Loss of trabecular structure; poor/no definition between cortical and medullary bone; increased density of long bones with defective modeling; metaphyseal under-modeling; transverse alternating bands of greater and lesser bone density in tubular bones; short femoral neck, increased width of the ribs; anteriorly notched vertebral bodies of the thoracolumbar spine; bone-in-bone appearance; severe bone marrow insufficiency
Ectodermal dysplasia and immunodeficiency 1 (EDAID1)	#300291	*IKBKG* (hemizygote, loss-of-function)	XLR Affecting only males	This gene encodes the regulatory subunit of the inhibitor of kappa B kinase complex, which activates the NF-kappa B transcription factor.	Osteopetrosis
Osteopetrosis and infantile neuroaxonal dystrophy	600329	Unknown	Not applicable	Not applicable.	Infantile severe osteopetrosis, an OPTB1-like bone phenotype
Dysosteoscleroris (DSS)	#224300	*SLC29A3* (homozygote or compound heterozygote missense, loss-of-function)	AR	This gene encodes a nucleoside transporter.	Osteosclerosis and platyspondyly; sclerotic calvaria, skull base, and long bones; flattened, deformed, and diffusely dense vertebral bodies; short stature and tendency to multiple fragility fractures, from infancy; oligodontia and delayed eruption of primary teeth
Pycnodysostosis (PYCD)	#265800	*CTSK* (homozygote or compound heterozygote, loss-of-function)	AR	This gene encodes cathepsin K, a potent endoprotease secreted by active osteoclasts to degrade the protein components of bone matrix.	Generalized bone mass and osteosclerosis; bone fragility (stress fractures of the tibia and femur, spondylolysis); short stature; deformity of the skull (including wide sutures); maxilla and mandible (obtuse angle of mandible) and phalanges (acro-osteolysis and short terminal phalanges); clavicular dysplasia
**1b. Diseases Characterized by High Bone Resorption (Increased Osteoclast Function)**					
Diffuse cystic angiomatosis of bone	123880	Unknown	Not applicable	Not applicable.	Early-onset progressive osteolysis caused by excessive bone resorption (monostotic or polyostotic occurrence), leading to skeletal deformities, functional impairment, and fragility fractures; localized bone pain
Juvenile-onset Paget’s disease of bone 5 (PDB5)	#239000	*TNFRSF11B* (homozygote or compound heterozygote, loss-of-function)	AR	This gene encodes osteoprotegerin (OPG), the soluble antagonist of RANK receptor signaling that inhibits osteoclastogenesis.	Short stature; progressive bone deformities (expanded bones, bowed long bones); fragile bones; pathological fractures; vertebral collapse; skull enlargement; and hyperostosis with progressive deafness, starting from infancy or childhood
Familial expansile osteolysis (FEO)	#174810	*TNFRSF11A* (heterozygote, gain-of-function)	AD	This gene encodes the RANK receptor, expressed on the membrane of osteoclast precursors and one of the main positive regulators of osteoclastogenesis.	Increased bone remodeling with osteolytic lesions, mainly affecting the appendicular skeleton; progressive osteoclastic high resorption leading to medullary and cortical expansion of the bone without sclerosis, accompanied by painful and disabling deformities and a tendency to pathologic fracture; premature loss of teeth
Early-onset Paget’s disease of bone 2 (PDB2)	#602080	*TNFRSF11A* (heterozygote, gain-of-function)	AD	This gene encodes the RANK receptor, expressed on the membrane of osteoclast precursors and one of the main positive regulators of osteoclastogenesis.	Focal abnormalities of bone segments (monostotic or polyostotic), mainly in the axial skeleton; skeletal pain and bony deformities of the lower limbs, such as bone enlargement and bowing of the long bones; skull can be affected by swelling and deformity of the jaw associated with loosening and loss of teeth, and progressive hearing loss; molecular evidence of increased osteoclastic bone resorption and disorganized bone structure at the lesions
Winchester syndrome	#277950	*MMP14* (homozygote, gain-of-function)	AR	This gene encodes a protein of the matrix metalloproteinase (MMP) family, involved in the degradation of various components of the extracellular matrix such as collagen, and essential for pericellular collagenolysis and modeling of skeletal and extraskeletal connective tissues during development. MMP14 is a target of PTH signaling in osteocytes, controlling bone resorption by regulating soluble RANKL secretion [15].	Generalized osteopenia/osteoporosis and bone thinning, leading to brittle bones, more prone to fracture; severe focal osteolysis starting in the hands and feet, causing pain and limiting movement; bone abnormalities later spread to other parts of the body, with joint problems (arthropathy) occurring in the elbows, shoulders, knees, hips, and spine
Hajdu-Cheney syndrome	#102500	*NOTCH2* (heterozygote, gain-of-function)	AD	This gene encodes a member of the Notch transmembrane protein family, controlling cell fate decisions. NOTCH2 has been reported to exhibit a stimulatory effect on osteoclastogenesis [16].	Generalized osteoporosis; progressive focal bone destruction including (acro-osteolysis); short stature; coarse and dysmorphic facies; bowing of the long bones; vertebral anomalies; dental anomalies; multiple fractures of the skull have been reported
**1c. Diseases Characterized by Low Bone Formation (Reduced Osteoblast Function and/or Matrix Mineralization)**					
Osteoporosis-pseudoglioma syndrome (OPPG)	#259770	*LRP5* (homozygote or compound heterozygote, loss-of-function)	AR	This gene encodes the LRP5 protein, which is expressed in osteoblast lineage, where it transduces the Wnt signaling, via the canonical pathway, promoting osteoblastogenesis.	Severe osteoporosis and dramatic reduction of trabecular bone, leading to major skeletal deformities and multiple pathological fractures; short stature (dwarfism in some cases); kyphoscoliosis
Cleidocranial dysplasia (CCD)	#119600	*RUNX2* (heterozygote, loss-of-function)	AD	This gene encodes the transcription factor RUNX2, a major positive regulator of the commitment of mesenchymal precursors to the osteoblast lineage.	Persistently open skull sutures with bulging calvaria; aplastic or hypoplastic clavicles; wide pubic symphysis, short ribs, short middle phalanx of the fifth fingers; dental anomalies; frequent vertebral malformation; supernumerary teeth; short stature
Multicentric osteolysis, nodulosis, and arthropathy (MONA)	#259600	*MMP2* (homozygote or compound heterozygote, loss-of-function)	AR	This gene encodes the matrix metalloprotease 2 (MMP2), which plays a crucial role in forming and maintaining the osteocytic canalicular network formation, a determinant of bone remodeling and mineralization [17].	Generalized mild to moderate osteoporosis; cortical thinning; increased caliber of the tubular and long bones; painful osteolysis of carpal and tarsal bones, associated with interphalangeal and metacarpophalangeal joint erosions
Bone mineral density quantitative trait locus 18 (BMND18)	#300910	*PLS3* (hemizygote, loss-of-function)	XLD	This gene encodes a protein involved in the formation of filamentous actin (F-actin) bundles, which are important for human bone health.	Early-onset osteoporosis and osteoporotic fractures
*Osteogenesis imperfecta* type V (OI5)	#610967	*IFITM5* (heterozygote, loss-of-function)	AD	This gene encodes a membrane protein thought to play a role in bone mineralization.	Moderate-severe form of OI.; bone fragility and low bone mass; calcification of the forearm interosseous membrane; radial head dislocation; hyperplastic callus formation; metaphyseal radiodense bands adjacent to growth plate (distal femur, proximal tibia, distal radius)
*Osteogenesis imperfecta* type VI (OI6)	#613982	*SERPINF1* (homozygote, loss-of-function)	AR	This gene encodes a protein involved in the correct regulation of mineralization.	Severe form of OI.; increased amount of unmineralized osteoid; severe hypomineralization; multiple fractures at birth and in the infancy; vertebral compression fractures; and deformities and fractures of long bones
*Osteogenesis imperfecta* type XII (OI12)	#613849	*SP7* (homozygote, loss-of-function)	AR	This gene encodes a zinc-finger transcription factor required for osteoblast differentiation and bone formation.	Moderate form of OI.; bone fragility and low bone mass; generalized osteoporosis; mild bone deformities; recurrent fractures in infancy; delayed tooth eruption; progressive hearing loss; short stature
*Osteogenesis imperfecta* type XV (OI15)	#615220	*WNT1* (homozygote or compound heterozygote, loss-of-function)	AR	This gene encodes a ligand of the canonical Wnt pathway, which is involved in the regulation of osteoblastogenesis.	Severe form of OI.; early-onset recurrent fractures; bone deformities; significant reduction of bone density; short stature; tooth development and hearing are normal
*Osteogenesis imperfecta* type XX (OI20)	#618644	*MESD* homozygote, loss-of-function)	AR	This gene encodes an endoplasmic reticulum-located chaperone protein, which is necessary for the receptors LRP5 and LRP6 of the canonical Wnt signaling and osteoblastogenesis [18].	Severe progressive form of OI.; many patients die due to respiratory failure in infancy, childhood or adolescence; progressive deforming bone dysplasia; severe osteopenia, skeletal deformities, and both healed and new multiple fractures on radiography (prenatal occurrence of fractures has been reported)
**1d. Diseases Characterized by High Bone Formation (Increased Osteoblast Function and/or Matrix Mineralization)**					
Metaphyseal dysplasia with maxillary hypoplasia with or without brachidactyly (MDMHB)	#156510	*RUNX2* (heterozygote, gain-of-function)	AD	This gene encodes the transcription factor RUNX2, a major positive regulator of the commitment of mesenchymal precursors to the osteoblast lineage.	Metaphyseal flaring of long bones; enlargement of the medial halves of the clavicles; maxillary hypoplasia; variable brachydactyly; dystrophic teeth
Osteopetrosis autosomal dominant 1 (OPTA1)	#607634	*LRP5* (heterozygote, gain-of-function)	AD	This gene encodes the LRP5 protein that is expressed in osteoblast lineage, where it transduces the Wnt signaling via the canonical pathway, promoting osteoblastogenesis.	Generalized osteosclerosis, most pronounced in the cranial vault; bone pain and hearing loss manifest in some cases; the only osteopetrosis disease that appears not to be associated with increased fracture rate
Autosomal dominant endosteal hyperostosis	#144750	*LRP5* (heterozygote, gain-of-function)	AD	This gene encodes the LRP5 protein that is expressed in osteoblast lineage, where it transduces the Wnt signaling via the canonical pathway, promoting osteoblastogenesis.	Cortical thickening of the long bones, with remarkable resistance of bone to fracture; osseous prominence of hard palate (torus palatinus); mandible elongation; forehead flattening
Van Buchen disease (hyperostosis corticalis generalisata, autosomal recessive endosteal hyperostosis)	#239100	17q21.31 Locus	AR	The 52-kb deletion approximately 35 kb downstream of the SOST gene, which removes a SOST-specific regulatory element.	Progressive skeletal overgrowth; cortical thickening; increased bone strength; osteosclerosis of the skull, mandible, clavicles, ribs, and diaphysis of the long bones beginning during puberty
Autosomal dominant craniodiaphyseal dysplasia (CDD)	#122860	*SOST* (heterozygote, loss-of-function)	AD	This gene encodes sclerostin, an inhibitor of mature osteoblast activity, inducing their transformation into inactive osteocytes.	Severe bone dysplasia characterized by massive, generalized hyperostosis and sclerosis, especially involving the skull and facial bones (leonine facies, progressive stenosis of craniofacial foramina)
Sclerostosis 1 (SOST1)	#269500	*SOST* (homozygote, loss-of-function)	AR	This gene encodes sclerostin, an inhibitor of mature osteoblast activity, inducing their transformation into inactive osteocytes.	Severe sclerosing bone dysplasia characterized by progressive skeletal overgrowth; syndactyly is a variable manifestation
Sclerostosis 2 (SOST2)	#614305	*LRP4* (heterozygote or homozygote, loss-of-function)	AD or AR	This gene encodes a member of the low-density lipoprotein receptor-related protein family, which is a regulator of Wnt signaling. Loss of LRP4 in osteoblast lineage cells increases bone formation and bone mass [19].	Severe sclerosing bone dysplasia characterized by progressive skeletal overgrowth; syndactyly is a variable manifestation
Osteopathia striata with cranial sclerosis	#300373	*AMER1* (hemizygote, loss-of-function)	XLD	This gene encodes a protein that acts as a negative regulator of Wnt signaling in osteoblast differentiation.	Fetal or neonatal lethality in males; in females. Sclerosis of the long bones and skull, macrocephaly, cleft palate; longitudinal striations of the long bones, pelvis, and scapulae
Buschke-Ollendorff syndrome (osteopoikilosis with or without melorheostosis)	#166700	*LEMD3* (heterozygote, loss-of-function)	AD	This gene encodes a protein involved in the regulation of both bone morphogenetic protein and TGFβ signaling.	Some individuals have bone manifestations (osteosclerotic foci in epimetaphyseal regions of the long bones); some individuals may also have melorheostosis, characterized by ‘flowing’ hyperostosis of the cortex of tubular bones
Craniometaphyseal dysplasia autosomal dominant (CMAD)	#123000	*ANKH* (heterozygote, loss-of-function)	AD	This gene encodes a transmembrane pyrophosphate transporter that channels intracellular pyrophosphate into the extracellular matrix, where it acts as inhibitor of mineralization.	Hyperostosis and sclerosis of the craniofacial bones associated with abnormal modeling of the metaphyses
Craniometaphyseal dysplasia autosomal recessive (CMAR)	#218400	*GJA1* (homozygote, gain-of-function)	AR	This gene encodes connexin 43, that permits coupling of osteoblasts and osteocytes, promoting bone formation.	Hyperostosis and sclerosis of the craniofacial bones associated with abnormal modeling of the metaphyses; sclerosis of the skull, leading to asymmetry of the mandible and cranial nerve compression with hearing loss and facial palsy
Camurati-Engelmann disease (CAEND)	#131300	*TGFβ1* (heterozygote, loss-of-function)	AD	This gene encodes the TGFβ1 protein that enhances osteoblast proliferation and production of matrix proteins during the early stages of osteoblast differentiation, blocks osteoblast apoptosis, and recruits osteoblastic precursors to the bone site through chemotactic attraction [20].	Cortical thickening of the diaphyses of the long bones; hyperostosis is bilateral and symmetrical and usually starts during childhood, at the diaphyses of the femora and tibiae, expanding to fibulae, humeri, ulnae, and radii; limb pain and sclerotic changes at the skull base may be present

OMIM, Online Mendelian Inheritance in Man^®^; “#” before the OMIM number indicates a confirmed Mendelian clinical phenotype with identified causative gene(s); no symbol before the OMIM number indicates a clinical phenotype for which the Mendelian basis, although suspected, has not been clearly established; AD, autosomal dominant; AR, autosomal recessive; XLD, X-linked dominant; XLR, X-linked recessive; RANK, receptor activator of nuclear factor κB; RANKL, receptor activator of nuclear factor κB ligand; OPG, osteoprotegerin; OI, *osteogenesis imperfecta*.

**Table 2:** Congenital metabolic bone disorders due to altered extracellular matrix proteins (disorders of collagen type 1 synthesis and assembly).

Disease Name	OMIM Phenotype Number	Causative Gene (Type of Mutations)	Inheritance	Gene Role	Reported Skeletal Phenotypes
**2a. Diseases due to Defect in Collagen Type 1 Synthesis and Structure**					
*Osteogenesis imperfecta* type I (OI1)	#166200	*COL1A1* (heterozygote, loss-of-function)	AD	This gene encodes the α1 chains of collagen type 1.	Mildest form of OI, due to a 50% reduction of the amount of collagen type 1; multiple bone fractures, usually resulting from minimal trauma, rare in the neonatal period and a constant onset starting from childhood to puberty, fracture rate decreases in the adulthood and often increases following menopause in women and after the sixth decade in men; general growth deficiency, but no remarkable craniofacial deformity; hearing loss occurs in about 50% of families; the subtype IA also presents dentinogenesis imperfecta, while the subtype IB has normal dentinogenesis
*Osteogenesis imperfecta* type II (OI2)	#166210	*COL1A1* (heterozygote, loss-of-function) or *COL1A2* (heterozygote, loss-of-function)	AD	*COL1A1* gene encodes the α1 chains of collagen type 1, while *COL1A2* gene encodes the α2 chain of collagen type 1.	Most severe form of OI.; perinatally lethal, due to rib cab deformity and respiratory insufficiency, following a premature birth; intrauterine fractures and abnormal skeletal modeling; severe hypomineralization of the skull bones (wide-open anterior and posterior fontanels); multiple neonatal fractures, severe bowing of long bones, severe undermineralization
*Osteogenesis imperfecta* type III (OI3)	#259420	*COL1A1* (heterozygote, loss-of-function) or *COL1A2* (heterozygote, loss-of-function)	AD	*COL1A1* gene encodes the α1 chains of collagen type 1, while *COL1A2* gene encodes the α2 chain of collagen type 1.	Severe form of OI, progressively deforming with age; pronounced growth impairment and craniofacial deformities, due to bending of head bones; dentinogenesis imperfecta; severe osteoporosis with multiple fractures starting from the infancy; progressive deformities of long bones and spine
*Osteogenesis imperfecta* type IV (OI4)	#166220	*COL1A1* (heterozygote, loss-of-function) or *COL1A2* (heterozygote, loss-of-function)	AD	*COL1A1* gene encodes the α1 chains of collagen type 1, while *COL1A2* gene encodes the α2 chain of collagen type 1.	Moderate form of OI.; pronounced growth impairment and craniofacial deformities, due to bending of head bones; osteoporosis with bone fractures; short stature; vertebral deformities and scoliosis; chronic bone pain; dentinogenesis imperfecta in some cases (OI type IVA)
*Osteogenesis imperfecta* type XVI (OI16)	#616229	*CREB3L1* (homozygote, loss-of-function) Mildly affected heterozygous family members have been reported	AR	This gene encodes a transcription factor essential for collagen production by osteoblasts during bone formation.	Most severe form of OI. Lethal; severe demineralization; prenatal and postnatal onset of multiple fractures of ribs and long bones; blue decreased ossification of the skull; heterozygous family members may exhibit osteopenia and recurrent fractures with minimal trauma
*Osteogenesis imperfecta* type XVII (OI18)	#617952	*TENT5A* (homozygote, loss-of-function)	AR	This gene encodes a cytoplasmic poly(A) polymerase, induced during osteoblast differentiation when it polyadenylates COL1A1 and COL1A2 mRNAs, increasing expression of both collagen type 1 α1 and α2 chains [24].	Severe form of OI.; congenital bowing of the long bones (femur and tibia); vertebral collapse; multiple fractures in the first years of life; poor mineralization; thin cortical bone; wormian bones at birth
**2b. Diseases due to Defect in Collagen Type 1 Post-Translational Modifications**					
*Osteogenesis imperfecta* type VII (OI7)	#610682	*CRTAP* (homozygote or compound heterozygote, loss-of-function)	AR	This gene encodes a cartilage-associated protein that, together with P3H1 and cyclophilin B, forms the collagen prolyl 3-hydroxylation complex in the endoplasmic reticulum. This complex is responsible for the modification of a single proline residue, Pro986, on the α1 chain of collagen type 1 [25].	Most severe form of OI.; early death for respiratory insufficiency; impaired growth; generalized severe osteoporosis, bone deformities at spine and long bones; and severe fractures at birth and during infancy; decreased cortical width and trabecular number; increased bone turnover; the long bones are characterized by a lack of diaphyseal modeling (undertubulation); short stature
*Osteogenesis imperfecta* type VIII (OI8)	#610915	*P3H1* (homozygote or compound heterozygote, loss-of-function)	AR	This gene encodes a collagen-prolyl-hydroxylases that, together with CRTAP and cyclophilin, forms the collagen prolyl 3-hydroxylation complex in the endoplasmic reticulum. This complex is responsible for the modification of a single proline residue, Pro986, on the α1 chain of collagen type 1 [25].	Most severe form of OI. lethal; severe growth deficiency (shortened long bones); extreme skeletal undermineralization; soft skull with wide open fontanel and bulbous metaphyses; severe osteoporosis with multiple fractures at birth
*Osteogenesis imperfecta* type IX (OI9)	#259440	*PPIB* (homozygote or compound heterozygote, loss-of-function)	AR	This gene encodes a protein located in the endoplasmic reticulum that concurs to the cis-trans isomerization of proline residues for proper folding of collagen fibrils.	Most severe form of OI.; early severe osteoporosis; severe bone undermineralization; multiple fractures at birth and in the infancy; severe deformities of long bones; molecular analysis of bone biopsies show an overhydroxylation of collagen type 1 components, over the entire length of the collagen and procollagen triple helix
**2c. Diseases due to Defect in Collagen Type 1 Processing and Crosslinking**					
*Osteogenesis imperfecta* type X (OI10)	#613848	*SERPINH1* (homozygote, loss-of-function)	AR	This gene encodes a collagen-binding protein that functions as a chaperone in the endoplasmic reticulum.	Severe form of OI.; generalized osteopenia/osteoporosis that causes multiple bone deformities and fractures in the early infancy (upper and lower extremities and ribs); dentinogenesis imperfecta
*Osteogenesis imperfecta* type XI (OI11)	#610968	*FKBP10* (homozygote or compound heterozygote, loss-of-function)	AR	This gene encodes a chaperone protein that participates in the correct folding of the procollagen type 1.	Severe form of OI.; progressive deforming oi. bones show a distorted lamellar structure and a fish scale-like pattern at the histological level; dentinogenesis imperfecta
Bruck syndrome type 1	#259450	*FKBP10* (homozygote, loss-of-function)	AR	This gene encodes a chaperone protein that participates in the correct folding of the procollagen type 1.	Severe form of OI bone phenotype; multiple fractures in infancy or early childhood; postnatal short stature; severe limb deformity; progressive scoliosis; congenital joint contractures (elbows and knees)
Bruck syndrome type 2	#609220	*PLOD2* (homozygote, loss-of-function)	AR	This gene encodes the lysyl hydroxylase 2 (LH2), which catalyzes the hydroxylation of the lysin residues of procollagen type 1 to hydroxylysine, necessary for the formation of covalent cross-links and collagen glycosylation.	Severe form of OI bone phenotype; multiple fractures in infancy or early childhood; postnatal short stature; severe limb deformity; progressive scoliosis; congenital joint contractures (elbows and knees)
*Osteogenesis imperfecta* type XIII (OI13)	#614856	*BMP1* (homozygote or compound heterozygote, loss-of-function)	AR	This gene encodes a bone morphogenetic protein that cleaves the procollagen type 1, yielding triple-helical molecules that associate into collagen type 1 fibrils.	Severe form of OI.; severe growth deficiency; borderline osteoporosis; severe bone deformities; multiple fractures (reported an average of 10 to 15 fractures per year) affecting both upper and lower limbs
*Osteogenesis imperfecta* type XIV (OI14)	#615066	*TMEM38B* (homozygote, loss-of-function)	AR	This gene encodes an endoplasmic reticulum membrane monovalent cation channel, controlling the intracellular calcium levels, thus, regulating multiple collagen-specific chaperones and modifying enzymes [26].	Severe form of OI.; variable degrees of severity of osteopenia and occurrence of multiple fractures, ranging from prenatal onset to 6 years of age; tooth development and hearing are normal
*Osteogenesis imperfecta* type XVII (OI17)	#616507	*SPARC* (homozygote, loss-of-function)	AR	This gene encodes osteonectin, an extracellular matrix protein that regulates processing of procollagen and collagen type 1 fibrillogenesis and incorporation into the extracellular matrix [27].	Severe form of OI.; short stature; kyphoscoliosis; general osteopenia,;long bone deformities (bowing); perinatal multiple fractures (multiple vertebral compression fractures of the thoracic and lumbar spine); infantile and childhood multiple fractures of long bones
*Osteogenesis imperfecta* type XIX (OI19)	#301014	*MBTPS2* (hemizygote, loss-of-function)	XLR	This gene encodes site-2 metalloprotease (S2P), located in the Golgi membrane, which cleaves procollagen type 1, favoring collagen assembly and crosslinking [28].	Moderate-severe form of OI.; generalized osteopenia; prenatal fractures; severe short stature in adulthood; variable scoliosis and pectal deformity; marked anterior angulation of the tibia
*Osteogenesis imperfecta* type XXI (OI21)	#619131	*KDELR2* (homozygote, loss-of-function)	AR	This gene encodes an endoplasmic reticulum protein retention receptor that binds HSP47, the chaperon protein encoded by the *SERPINH1* gene, inducing its dissociation from collagen type 1. In the absence of KDELR2 protein or in the presence of mutated/inactive protein, HSP47 remains bound to collagen molecules extracellularly, disrupting fiber formation [29].	Severe form of OI.; progressively deforming bone disorder (long bone bowing); multiple fractures (long bone fractures, vertebral compression, platyspondyly), occurring after minor trauma, manifesting from early childhood or being present at birth in some cases; disproportionate short stature and scoliosis; patients are often wheelchair-bound by adulthood
Cole-Carpenter syndrome type 1 (CLCRP1)	#122240	*P4HB* (heterozygote, loss-of-function)	AD	This gene encodes the β chain of the prolyl 4-hydroxylase, which forms a tetrameric complex with two P4HAs (α chain) to form the active prolyil 4-hydroxylase, which catalyzes the hydroxylation of proline residues in pre-procollagen type 1 molecules.	An OI-like bone disorder; osteopenia; bone fragility (bone deformities and multiple fractures); craniosynostosis; distinct facial features (frontal and temporal bossing of the skull, ocular proptosis, micrognathia, high-arched palate, low-set ears)
Cole-Carpenter syndrome type 2 (CLCRP2)	#616294	*SEC24D* (compound heterozygote, loss-of-function)	AR	This gene encodes a protein belonging to the SEC23/SEC24 family, which is involved in vesicle trafficking. SEC24D is specifically important for collagen secretion from the endoplasmic reticulum [30].	An OI-like bone disorder; osteopenia; thin bones; bone fragility (bone deformities at long bones, and postnatal fractures); craniosynostosis; hydrocephalus; distinctive facial features (marked frontal bossing, ocular proptosis, high palate, midface hypoplasia, micrognathia); wormian bones; short stature

OMIM, Online Mendelian Inheritance in Man^®^; “#” before the OMIM number indicates a confirmed Mendelian clinical phenotype with identified causative gene(s); AD, autosomal dominant; AR, autosomal recessive; XLR, X-linked recessive; OI, *osteogenesis imperfecta*.

**Table 3:** Congenital metabolic bone disorders due altered bone microenvironmental regulators.

Disease Name	OMIM Phenotype Number	Causative Gene (Type of Mutations)	Inheritance	Gene Role	Reported Skeletal Phenotypes
**3a. Diseases due to Defect of Alkaline Phosphatase Activity**					
Hypophosphatasia, perinatal lethal (HPPN)	#241500 (ORPHA 247623)	*ALPL* (homozygote or compound heterozygote, loss-of-function)	AR	This gene encodes for the tissue non-specific alkaline phosphatase (TNALP) protein.	Lethal form of HPP. Severe uterine generalized bone hypomineralization, causing stillbirth or lethal respiratory failure within days of birth
Hypophosphatasia, prenatal benign (HPPPB)	(ORPHA 247638)	*ALPL* (heterozygote, homozygote or compound heterozygote, loss-of-function)	AD or AR	This gene encodes for the tissue non-specific alkaline phosphatase (TNALP) protein.	Benign form of HPP. Prenatal skeletal manifestations (limb shortening and bowing) that slowly resolve spontaneously; bone improvements are reported from the third trimester of pregnancy and after birth; later in life, disease may develop into moderate childhood HPP, adult HPP, or odonto-HPP
Hypophosphatasia, infantile (HPPI)	#241500 (ORPHA 247651)	*ALPL* (homozygote or compound heterozygote, loss-of-function)	AR	This gene encodes for the tissue non-specific alkaline phosphatase (TNALP) protein.	Severe form of HPP; severe bone hypomineralization, causing infantile rickets (onset between birth and six months of age); softening or thinning of the skull; craniosynostosis; rachitic ribs; scoliosis; thickening of wrists and ankles; bowing of long bones. growth failure, short stature; many affected patients are at risk of respiratory failure, within the first year of life, due to rachitic deformities of the rib cage
Hypophosphatasia, childhood (HPPC)	#241510 (ORPHA 247667)	*ALPL* (heterozygote, homozygote or compound heterozygote, loss-of-function)	AD or AR	This gene encodes for the tissue non-specific alkaline phosphatase (TNALP) protein.	Moderate form of HPP; onset of skeletal manifestation after six months and, generally, before five years of age; widely variable clinical features, i.e., low bone mineral density, rickets, skeletal deformities, pathological fractures, short stature
Hypophosphatasia, adult (HPPA)	#146300 (ORPHA 247676)	*ALPL* (heterozygote, homozygote or compound heterozygote, loss-of-function)	AD or AR	This gene encodes for the tissue non-specific alkaline phosphatase (TNALP) protein.	Mild form of HPP; widely variable clinical features, i.e., adult onset osteomalacia, early-onset osteoporosis, chondrocalcinosis, osteoarthropathy, musculoskeletal pain, stress fractures of metatarsal bones and tibia, femoral pseudofractures, dental anomalies, recurrent caries, loss of permanent dentition
Odontohypophosphatasia	#146300 (ORPHA 247685)	*ALPL* (heterozygote, homozygote or compound heterozygote, loss-of-function) 74% of cases are heterozygotes	AD or AR	This gene encodes for the tissue non-specific alkaline phosphatase (TNALP) protein.	Mild form of adult HPP; only teeth are affected; no bone clinical manifestations; premature exfoliation of fully rooted deciduous teeth and/or severe dental caries
**3b. Diseases due to Alterations of the RANK/RANKL/OPG System**					
Osteopetrosis autosomal recessive type 2 (OPTB2)	#259710	*TNFSF11* (homozygote, loss-of-function)	AR	This gene encodes RANKL, the ligand of RANK that induces osteoclastogenesis.	Mandibular osteomyelitis and prognathism; dental anomalies; metaphyseal modeling defects; bone deformities; poor bone remodeling; tendency to fracture
Osteopetrosis autosomal recessive type 7 (OPTB7)	#612301	*TNFRSF11A* (homozygote or compound heterozygote, loss-of-function)	AR	This gene encodes the RANK receptor, expressed on the membrane of osteoclast precursors and one of the main positive regulators of osteoclastogenesis.	Severe osteoclast-poor osteopetrosis; extensive trabecular structures, with retention of large areas of cartilage
Familial expansile osteolysis (FEO)	#174810	*TNFRSF11A* (heterozygote, gain-of-function)	AD	This gene encodes the RANK receptor, expressed on the membrane of osteoclast precursors and one of the main positive regulators of osteoclastogenesis.	Increased bone remodeling with osteolytic lesions, mainly affecting the appendicular skeleton; progressive osteoclastic high resorption leading to medullary and cortical expansion of the bone without sclerosis, accompanied by painful and disabling deformities and a tendency to pathologic fracture; premature loss of teeth
Early-onset Paget’s disease of bone 2 (PDB2)	#602080	*TNFRSF11A* (heterozygote, gain-of-function)	AD	This gene encodes the RANK receptor, expressed on the membrane of osteoclast precursors and one of the main positive regulators of osteoclastogenesis.	Focal abnormalities of bone segments (monostotic or polyostotic), mainly in the axial skeleton; skeletal pain and bony deformities of the lower limbs, such as bone enlargement and bowing of the long bones; skull can be affected with swelling and deformity of the jaw associated with loosening and loss of teeth, and progressive hearing loss; molecular evidence of increased osteoclastic bone resorption and disorganized bone structure at the lesions
Juvenile-onset Paget’s disease of bone 5 (PDB5)	#239000	*TNFRSF11B* (homozygote or compound heterozygote, loss-of-function)	AR	This gene encodes osteoprotegerin (OPG), the soluble antagonist of RANK receptor signaling that inhibits osteoclastogenesis.	Short stature; progressive bone deformities (expanded bones, bowed long bones); fragile bones; pathological fractures; vertebral collapse; skull enlargement; hyperostosis with progressive deafness, starting from infancy or childhood
**3c. Diseases due to Alterations of the Glycosylphosphatidylinositol Biosynthesis Pathway**					
Hyperphosphatasia with mental retardation syndrome 1 (HPMRS1)	#239300	*PIGV* (homozygote or compound heterozygote, loss-of-function)	AR	This gene encodes a protein involved in the biosynthesis of GPI by adding the second mannose residue to the GPI core.	Hyperphosphatasia; facial dysmorphism; variable degrees of brachytelephalangy
Hyperphosphatasia with mental retardation syndrome 2 (HPMRS2)	#614749	*PIGO* (compound heterozygote, loss-of-function)	AR	This gene encodes a protein involved in the biosynthesis of GPI by transferring a of phosphatidylethanolamine to the third mannose residue of the GPI core.	Hyperphosphatasia; moderately to severely delayed psychomotor development; facial dysmorphism; brachytelephalangy
Hyperphosphatasia with mental retardation syndrome 3 (HPMRS3)	#614207	*PGAP2* (homozygote or compound heterozygote, loss-of-function)	AR	This gene encodes a protein involved in the lipid remodeling steps of GPI-anchor maturation.	Hyperphosphatasia; very poor motor development; cleft palate
Hyperphosphatasia with mental retardation syndrome 4 (HPMRS4)	#615716	*PGAP3* (homozygote or compound heterozygote, loss-of-function)	AR	This gene encodes a GPI-specific phospholipase A2, involved in fatty acid GPI remodeling.	Hyperphosphatasia; delayed psychomotor development; dysmorphic facial features (hypertelorism, upslanting palpebral fissures, broad nasal bridge, short nose, long philtrum)
Hyperphosphatasia with mental retardation syndrome 4 (HPMRS4)	#616025	*PIGW* (homozygote or compound heterozygote, loss-of-function)	AR	This gene encodes a protein in the biosynthesis of GPI, by the acylation of the inositol ring of phosphatidylinositol.	Hyperphosphatasia; lack of psychomotor development; facial dysmorphism manifests in some patients
Hyperphosphatasia with mental retardation syndrome 4 (HPMRS4)	#616809	*PIGY* (homozygote, loss-of-function)	AR	This gene encodes a protein that is part of the GPI-N-acetylglucosaminyltransferase complex which initiates the biosynthesis of GPI.	Hyperphosphatasia; global developmental delay; dysmorphic features (bitemporal narrowing, depressed nasal bridge with upturned nares, short neck); in some cases, brachytelephalangy, proximal limb shortening, hip dysplasia, and osteopenia have been reported
**3d. Diseases due to Alterations of the LRP5-Wnt Signaling**					
*Osteogenesis imperfecta* type XV (OI15)	#615220	*WNT1* (homozygote or compound heterozygote, loss-of-function)	AR	This gene encodes a ligand of the canonical Wnt pathway, involved in the regulation of osteoblastogenesis.	Severe form of OI.; early-onset recurrent fractures; bone deformities; significant reduction of bone density; short stature; tooth development and hearing are normal
*Osteogenesis imperfecta* type XX (OI20)	#618644	*MESD* (homozygote, loss-of-function)	AR	This gene encodes an endoplasmic reticulum-located chaperone protein, necessary for the receptors LRP5 and LRP6 of the canonical Wnt signaling and osteoblastogenesis [18].	Severe progressive form of OI.; several patients die due to respiratory failure in infancy, childhood, or adolescence; progressive deforming bone dysplasia; severe osteopenia; skeletal deformities; and both healed and new multiple fractures on radiography (prenatal occurrence of fractures has been reported)
Osteoporosis-pseudoglioma syndrome (OPPG)	#259770	*LRP5* (homozygote or compound heterozygote, loss-of-function)	AR	This gene encodes the LRP5 protein, which is expressed in osteoblast lineage, where it transduces the Wnt signaling, via the canonical pathway, promoting osteoblastogenesis.	Severe osteoporosis and dramatic reduction of trabecular bone, leading to major skeletal deformities and multiple pathological fractures; short stature (dwarfism in some cases), kyphoscoliosis
Osteopetrosis autosomal dominant 1 (OPTA1)	#607634	*LRP5* (heterozygote, gain-of-function)	AD	This gene encodes the LRP5 protein, which is expressed in osteoblast lineage, where it transduces the Wnt signaling via the canonical pathway, promoting osteoblastogenesis.	Generalized osteosclerosis, most pronounced in the cranial vault; bone pain and hearing loss manifest in some cases; the only osteopetrosis disease that appears not to be associated with increased fracture rate
Autosomal dominant endosteal hyperostosis	#144750	*LRP5* (heterozygote, gain-of-function)	AD	This gene encodes the LRP5 protein, which is expressed in osteoblast lineage, where it transduces the Wnt signaling via the canonical pathway, promoting osteoblastogenesis.	Cortical thickening of the long bones, with remarkable resistance of bone to fracture; osseous prominence of hard palate (torus palatinus); mandible elongation; forehead flattening
**3e. Diseases due to Alteration of the Bone Morphogenetic Proteins Receptor**					
Fibrodysplasia ossificans progressive (FOP)	#135100	*ACVR1* (heterozygote, gain-of-function)	AD	This gene encodes activin A, critical regulators of bone formation. Inappropriate activation of activin A, as in FOP, activates a pathological osteogenesis osteogenic in endothelial cells and soft tissues (ectopic calcification).	Progressive ossification of skeletal muscle, fascia, tendons, and ligaments, occurring as a consequence of an inevitable and unpredictable soft tissue low trauma

OMIM, Online Mendelian Inheritance in Man^®^; “#” before the OMIM number indicates a confirmed Mendelian clinical phenotype with identified causative gene(s); AD, autosomal dominant; AR, autosomal recessive; RANK, receptor activator of nuclear factor κB; RANKL, receptor activator of nuclear factor κB-ligand; OPG, Osteoprotegerin; OI, *osteogenesis imperfecta*; HPP, hypophosphatasia; GPI, glycosylphosphatidylinositol.

**Table 4:** Congenital metabolic bone disorders due to alterations of vitamin D metabolism and activity.

Disease Name	OMIM Phenotype Number	Causative Gene (Type of Mutations)	Inheritance	Gene Role	Reported Skeletal Phenotypes
Vitamin D hydroxylation-deficient rickets type 1A (VDDR1A)	#264700	*CYP27B1* (homozygote or compound heterozygote, loss-of-function)	AR	This gene encodes the 25-hydroxyvitamin D3-1-α-hydroxylase, an enzyme in the renal proximal tubule, which catalyzes the hydroxylation of 25-hydroxyvitamin D3 into the active form, 1,25(OH)2D3.	Growth retardation; congenital rickets; severe hypocalcemia leading to osteomalacia and rachitic bone deformations (long bone deformities); enlarged costochondral junctions of the ribs; pectus carinatum; metaphyseal flaring of wrists and ankles; frontal bossing; open fontanels; enlarged skull sutures
Vitamin D hydroxylation-deficient rickets type 1B (VDDR1B)	#600081	*CYP2R1* (homozygote or compound heterozygote, loss-of-function)	AR	This gene encodes the vitamin D 25-hydroxylase, which catalyzes the initial hydroxylation of vitamin D at carbon 25, in the liver.	Severe hypocalcemia leading to osteomalacia; rachitic bone deformations (bowing of long bones, lower limb deformities) and fractures; sparse bone trabeculae; thin bone cortex; distorted epiphyses, frayed and irregular metaphyses
Vitamin D-dependent rickets type 2A (VDDR2A)	#277440	*VDR* (homozygote or compound heterozygote, loss-of-function)	AR	This gene encodes the receptor of the vitamin D, which mediates the cell response to vitamin D signal.	Early-onset progressive severe rickets with hypocalcemia, manifests during the first year of life; poor growth; skeletal deformities (bowing of the femur, tibia, fibula, enlargement of the wrists and ankles); fracture and pseudofractures
Vitamin D-dependent rickets type 2B (VDDR2B)	%600785	Unknown	Non applicable	Non applicable.	Early-onset progressive severe rickets with hypocalcemia, manifests during the first year of life; poor growth; skeletal deformities (bowing of the femur, tibia, fibula, enlargement of the wrists and ankles); fracture and pseudofractures
Vitamin D-dependent rickets type 3 (VDDR3)	#619073	*CYP3A4* (heterozygote, gain-of-function)	AD	This gene encodes the cytochrome P450 3A4, the predominant P450 expressed in adult human liver, involved in the oxidative metabolism and catabolism of vitamin D.	Early-onset progressive severe rickets with hypocalcemia, manifests during the first year of life; poor growth; skeletal deformities; bowing of long legs

OMIM, Online Mendelian Inheritance in Man^®^; “#” before the OMIM number indicates a confirmed Mendelian clinical phenotype with identified causative gene(s); “%” before the OMIM number indicates a confirmed Mendelian phenotype for which the underlying genetic basis is still unknown; AD, autosomal dominant; AR, autosomal recessive.

**Table 5:** Congenital disorders of the phosphate homeostasis.

Disease Name	OMIM Phenotype Number	Causative Gene (Type of Mutations)	Inheritance	Gene Role	Reported Skeletal Phenotypes
**5a. Hypophosphatemic Disorders**					
Autosomal dominant hypophosphatemic rickets (ADHR)	#193100	*FGF23* (heterozygote, gain-of-function)	AD	This gene encodes the phosphotropic hormone FGF23, which decreases the reabsorption of phosphate at the kidney level, increasing phosphate excretion and reducing phosphate serum concentration.	ADHR shows incomplete penetrance; variable age at onset (childhood to adult); early onset (1–3 years of age), rickets, severe bowing of the extremities, enlarged costochondral junctions of the ribs; late onset (during puberty), bone pain; no bowing of the lower limbs
X-linked dominant hypophosphatemic rickets (XLHR)	#307800	*PHEX* (hemizygote, loss-of-function)	XLD	This gene encodes a Zn-metallo-endopeptidase that cleaves, and inactivates, full-length FGF23, blocking the FGF23-mediated positive regulation of renal phosphate excretion, and thus increases levels of circulating phosphate.	Rickets with bone deformities (bowing of lower extremities, enlarged costochondral junctions of the ribs, metaphyseal flaring of wrists and ankles, and frontal bossing); short stature; dental anomalies (late dentition and tooth abscesses)
Osteoglophonic dysplasia (OGD)	#166250	*FGFR1* (heterozygote, gain-of-function)	AD	This gene encodes the dominant FGF receptor (FGFR1) mediating the effects of FGF23 in proximal and distal renal tubules.	Severe rhizomelic dwarfism; rickets/osteomalacia; non-ossifying bone lesions; craniosynostosis; prominent supraorbital ridge; frontal bossing; depressed nasal bridge; prognathism
Hypophosphatemic rickets and hyperparathyroidism (HRH)	%612089	Unknown	Not applicable	Not applicable.	Rickets; prominent forehead; large open anterior fontanel; knobby wrists; moderately bowed legs
Autosomal recessive hypophosphatemic rickets type 1 (ARHR1)	#241520	*DMP1* (homozygote, loss-of-function)	AR	This gene encodes the dentin matrix acidic phosphoprotein 1, which is involved in the regulation of bone mineralization.	Rickets/osteomalacia; leg bowing; widening of the metaphyses; under-modelled ribs and clavicles; short stature; osteosclerosis of the base of the skull and the calvaria bones
Autosomal recessive hypophosphatemic rickets type 2 (ARHR2)	#613312	*ENPP1* (homozygote, loss-of-function)	AR	This gene encodes the ectonucleotide pyrophosphate/phosphodiesterase 1, an enzyme that hydrolyses inorganic pyrophosphate, eliminating its antimineralization action.	Rickets/osteomalacia; rib and clavicle anomalies; short stature; osteosclerosis of the base of the skull
X-linked recessive hypophosphatemic rickets (XLRHR)	#609826	*CLCN5* (hemizygote, loss-of-function)	XLR	This gene encodes a proton-coupled chloride transporter, which exchanges chloride ions against protons.	Males present with rickets or osteomalacia; hypophosphatemia; hypercalciuria
Hereditary hypophosphatemic rickets with hypercalciuria (HHRH)	#241530	*SLC34A3* (homozygote or compound heterozygote, loss-of-function)	AR	This gene encodes a sodium/phosphate cotransporter protein (NPTIIc), expressed primarily in the kidney, which concur to renal phosphate reabsorption.	Radiographic and/or histologic evidence of rickets/osteomalacia; bone pain; short stature; limb deformities; muscle weakness
Hypophosphatemia nephrolitiasis osteoporosis type 1 (NPHLOP1)	#612286	*SLC34A1* (heterozygote, loss-of-function)	AD	This gene encodes a sodium/phosphate cotransporter protein (NPTIIa), expressed primarily in the kidney, which concur to renal phosphate reabsorption.	Bone demineralization; osteoporosis; spinal deformities; fragility fractures
Hypophosphatemia nephrolitiasis osteoporosis type 2 (NPHLOP2)	#612287	*SLC9A3R1* (heterozygote, loss-of-function)	AD	This gene encodes a sodium/hydrogen exchanger regulatory cofactor (NHERF1) of the sodium/phosphate cotransporters, concurring to the renal phosphate reabsorption.	Decreased bone mineral density; osteoporosis; spinal deformities
**5b. Hyperphosphatemic Disorders**					
Hyperphosphatemic familial tumoral calcinosis type 1 (HFTC1)	#211900	*GALNT3* (homozygote or compound heterozygote, loss-of-function)	AR	This gene encodes an enzyme of the Golgi (ppGaNTase-T3) that is responsible for glycosylation and prevention of glycolysis of FGF23 protein, granting the activation of FGF23.	Progressive deposition of basic calcium phosphate crystals in periarticular spaces, soft tissues (ectopic multiple calcifications), and sometimes bone; in some cases, the disorder is characterized by involvement of the long bones associated with the radiographic findings of periosteal reaction and cortical hyperostosis (“hyperostosis-hyperphosphatemia syndrome”)
Hyperphosphatemic familial tumoral calcinosis type 2 (HFTC2)	#617993	*FGF23* (homozygote, loss-of-function)	AR	This gene encodes the phosphotropic hormone FGF23, which decreases the reabsorption of phosphate at the kidney level, increasing phosphate excretion and reducing phosphate serum concentration.	Progressive deposition of basic calcium phosphate crystals in periarticular spaces, soft tissues (ectopic multiple calcifications), and sometimes bone; in some cases, the disorder is characterized by involvement of the long bones associated with the radiographic findings of periosteal reaction and cortical hyperostosis (“hyperostosis-hyperphosphatemia syndrome”)
Hyperphosphatemic familial tumoral calcinosis type 3 (HFTC3)	#617994	*KLOTHO* (homozygote, loss-of-function)	AR	This gene encodes a co-receptor protein (KL) that increases the affinity of FGF23 for its receptors, favoring the FGF23 signaling.	Progressive ectopic calcifications; osteopenia; patchy sclerosis in the hands, feet, long bones, and calvaria; intracranial calcifications

OMIM, Online Mendelian Inheritance in Man^®^; “#” before the OMIM number indicates a confirmed Mendelian clinical phenotype with identified causative gene(s); “%” before the OMIM number indicates a confirmed Mendelian phenotype for which the underlying genetic basis is still unknown. AD, autosomal dominant; AR, autosomal recessive; XLD, X-linked dominant; XLR, X-linked recessive.

## Data Availability

Non applicable.
